# Validation of the Awareness Atlas—a new measure of the manifestation of consciousness

**DOI:** 10.3389/fpsyg.2024.1283980

**Published:** 2024-03-21

**Authors:** Yuane Jia, Margaret Schenkman, Hester O Connor, Krishnmurthy Jayanna, Rosalind Pearmain, Annelies Van’t Westeinde, Kamlesh D. Patel

**Affiliations:** ^1^Department of Interdisciplinary Studies, School of Health Professions, Rutgers University, Newark, NJ, United States; ^2^Department of Physical Medicine and Rehabilitation, Physical Therapy Program, University of Colorado, Aurora, CO, United States; ^3^Retired, Kildare, Ireland; ^4^Division of Public Health, Faculty of Life and Allied Health Sciences, Ramaiah University of Applied Sciences, Bangalore, India; ^5^Center for Integrative Health and Wellbeing, Bangalore, India; ^6^Minster Centre, London, United Kingdom; ^7^Department of Women’s and Children’s Health, Unit for Pediatric Endocrinology, Karolinska Institutet, Stockholm, Sweden; ^8^Heartfulness Institute, Kanha Shanti Vanam, Hyderabad, India

**Keywords:** scale development, validation, new measure, manifestation of consciousness, awareness, Heartfulness meditation

## Abstract

Consciousness has intrigued philosophers and scholars for millennia and has been the topic of considerable scientific investigation in recent decades. Despite its importance, there is no unifying definition of the term, nor are there widely accepted measures of consciousness. Indeed, it is likely that consciousness—by its very nature—eludes measurement. It is, however, possible to measure how consciousness manifests as a lived experience. Yet here, too, holistic measures are lacking. This investigation describes the development and validation of the Awareness Atlas, a measure of the manifestation of consciousness. The scale was informed by heart-based contemplative practices and the resulting lived experience with a focus on the impacts of manifestation of consciousness on daily life. Four hundred forty-nine individuals from the USA, Canada, India, and Europe participated in psychometric testing of the scale. Exploratory and confirmatory factor analyses were used for validation, demonstrating excellent validity in measuring manifestation of consciousness. The final model fit exceeded all required thresholds, indicating an excellent fitted model with a single dimensionality to measure the manifestation of consciousness comprised of four subscales: Relationship to Others; Listening to the Heart; Connection with Higher Self; and Acceptance and Letting Go. Number of years meditating and practicing Heartfulness meditation were positively related to the total and subscale scores. Test–retest reliability was excellent for the total scale, and good to excellent for the four subscales. Findings demonstrate that the Awareness Atlas is a well-constructed tool that will be useful in examining changes in manifestation of consciousness with various experiences (e.g., meditation, life-altering conditions).

## Introduction

Consciousness has intrigued philosophers and scholars for millennia and has been the focus of considerable scientific investigation in recent decades (Garcia-Castro and Kodukula, 2022). There is increasing interest in the role consciousness plays with respect to health and well-being (George et al., 2000; Bożek et al., 2020; Jayanna, 2021). Further, there is heightened realization that a shift in human consciousness is necessary to address global challenges such as societal conflicts, climate change, and global warming (Kirsmayer, 2010; Nasibulina, 2015). To these ends, various approaches have been employed in recent years to expand consciousness (e.g., educational, psychological, religious and spiritual) (Vieten et al., 2006; Thiengkamol, 2011; Savelyeva and Douglas, 2017; Patel, 2023).

Exploration of how consciousness manifests in daily life, and of intentional changes in consciousness, requires a spectrum of manifestations from the personal to the universal. Such explorations are limited by the lack of a simple measure that incorporates the range of manifestations of consciousness experienced by individuals. Against this backdrop, we believe research is necessary to develop and validate a tool to assess shifts in human consciousness.

### The problem of defining consciousness

#### Conceptualizing consciousness

Consciousness has been characterized in numerous ways without consensus (Guertin, 2019; Garcia-Castro and Kodukula, 2022). A few examples illustrate the diverse descriptions and resulting difficulties with measuring this construct.

Early discussions of consciousness come from Eastern traditions. For example, four states of consciousness were described in the Mandukya Upandishad (Srinivasan, 2022): wakefulness, dreaming, sleep, and the Turiya state, which transcends the first three states: “*perceiving neither what is inside nor what is outside… neither as perceiving nor as not perceiving… as unthinkable; as indescribable…*” [quoted from Olivelle (Srinivasan, 2022)]. The “state of pure consciousness” described in the Buddhist tradition appears to be similar to the Turiya state described in the Upanishads (Srinivasan, 2022). Heartfulness identifies a fifth state, the Turiyatit state, which extends the Turiya state experienced in meditation out into everyday life (Patel, 2019).

Consciousness can be considered to move along a spectrum from zero to infinity (Wilber, 2000; van’t Westeinde and Patel, 2022). Drawing from Patanjali and the Vedas, van’t Westeinde and Patel noted that the goal of yoga and other contemplative traditions, “Union with Universal Consciousness,” is accomplished through purifying and expanding individual consciousness to such an extent that it merges with the universal. Some investigators use the term “pure awareness” in the context of consciousness. For example, Gamma and Metzinger (2021) suggested that pure awareness is characterized by “an absence of space or time and body sense” and by the experience of “peacefulness” and “unboundedness.” These authors used the term “minimal phenomenal experience” to describe the condition. Thomas et al. (2022) suggested that “The original cause [of consciousness] is nothingness.” Some contemplative practices talk about pure consciousness as consciousness without content, while another term, “non-conceptual representational content,” has been proposed in the context of consciousness (Srinivasan, 2022).

In contrast, from a Western perspective, Kihlstrom (2022) suggested that consciousness has to do with monitoring ourselves and our environment, which is linked to voluntarily starting and ending both mental and behavioral activities. This monitoring allows for memory, thoughts, feelings, emotions, and desires to be represented in phenomenological awareness, suggesting that consciousness and self are inexorably intertwined.

Wilber recognized the importance of integrating Eastern philosophies with contemporary Western approaches to psychological development (Wilber, 2000). To this end, he drew on the work of a variety of scholars (e.g., Loevinger, 1976; Kegan, 1982) and attempted to integrate spiritual philosophies, adult developmental psychology (including development of moral reasoning and ethics), humanistic, transpersonal, and positive psychologies. His spectrum of consciousness explores a span from everyday consciousness to the Turyatit state of yogis (Wilber et al., 1986; Wilber, 2000).

This sample of descriptions above illustrates that consciousness encompasses a spectrum from simple awareness of self and others to pure consciousness that is without content or that has non-conceptual representational content. In other words, consciousness in its most refined state is beyond description. Clearly, there are significant and substantial challenges to measuring such an ill-defined construct, especially if one includes a state without content.

#### Manifestation of consciousness

An alternative and more feasible approach to understanding consciousness is to study its expression, which we refer to as the “manifestation of consciousness.” While consciousness itself cannot be easily defined, its effects on human behavior, thoughts, and feelings can be characterized. The awareness of the thoughts and feelings of oneself and others may grow as one experiences expansion of consciousness. This is one aspect of an expanding consciousness, and one that we will focus upon for this study. From this perspective, expanding consciousness can be considered as an expansion of one’s awareness of self and others. By examining the manifestation of consciousness, it is possible to identify relevant phenomena that can be measured quantitatively. This approach has been adopted across multiple disciplines, including medicine and neurology (Teasdale and Jennett, 1974; Sattin et al., 2021), psychology and behavior (Turner et al., 1978; Grant et al., 2002), sociology and human development (Carver and Glass, 1976; Diemer et al., 2017).

As with consciousness itself, the manifestation of consciousness is characterized differently depending on the discipline (Guertin, 2019). For example, anesthesiologists consider consciousness in terms of alertness and awareness when determining whether a patient is sufficiently “unconscious” to perform surgery. To determine the level of consciousness post traumatic brain injury, medical professionals typically assess eye opening, ability to speak, and ability to move on request (e.g., as measured with the Glasgow Coma Scale) (Teasdale and Jennett, 1974). Behavioral scientists can conceptualize and investigate changes in consciousness in the context of awareness/perception of self or others, with various interventions as a means of examining that aspect of increasing awareness or consciousness. Thus, studies to examine changing manifestations of consciousness (e.g., empathy) can be designed and used to draw inferences about changing states of consciousness with different situations, interventions, and life experiences. By examining the manifestation of consciousness in these contexts, it should be possible to begin to appreciate the practical role of expanding consciousness in daily life.

The above approaches do not address the expression of consciousness described in contemplative practices (mostly of Eastern origin) as “non-conceptual representational content,” “consciousness without content,” or “universality” (Srinivasan, 2022). Literature from transpersonal psychology focuses on consciousness from the perspective of self-transcendence, which comes closer to these concepts (MacDonald and Friedman, 2013; Kitson et al., 2020). Macdonald and Friedman (2013) suggested that transcendence is a state that can be appreciated, but cannot easily be put into words, and yet must be investigated through quantitative methods in order to study its impact. Kitson et al. (2020) pointed out that any investigation of self-transcendence also needs to take into account the discipline investigating its impact (e.g., psychology, gerontology, nursing) because manifestation of consciousness, like consciousness itself, is multi-faceted. Each discipline brings a different focus to the issues.

### Meditation and its impact on consciousness

Meditation and yoga have long been used as tools to change states of consciousness in Eastern spiritual traditions (e.g., as described in the Yoga Sutras of Patanjali) (Deshpande, 2021). In the recent past, studies have been undertaken to investigate the effects of meditation on states of mind and consciousness. The state and trait effects of meditation are found to have implications on the neuroscience of attention, consciousness, self-awareness, and empathy (Raffone and Srinivasan, 2010; Sparby, 2015; Tripathi and Bharadwaj, 2021).

During the 20th century, several meditation approaches based on ancient practices have emerged that are specifically focused on the heart (Lindhard, 2018a; van’t Westeinde and Patel, 2022; Patel 2023). A heart-based approach can lead a person through different levels of consciousness. Examples include the Arka Dhyana method, developed by Srinivas Arka, which has some similarities to Prayer of the Heart (Lindhard, 2017, 2018a,b) and Heartfulness Meditation (Patel and Pollock, 2018; Patel, 2023). Although each approach recognizes a role of the heart in the development and expression of different levels of consciousness, they have different theoretical frameworks and descriptions of the journey. For example, Arka Dhyana refers to stages of consciousness progressing through six levels from a heart-mind stage to a sixth stage of Pure Self, described as “consciousness without content.” The Arka Dhyana method uses developing awareness of intuitive feeling (as opposed to physical feeling) to travel this journey. The six levels convey a distinct separation of states.

In contrast, Heartfulness Meditation, a form of Raja Yoga, refers to a gradual expansion of consciousness through various layers or regions, which are uncovered by the removal of old impressions (referred to by the Sanskrit term *samskaras*), and awakened by the impulse of Transmission during meditation. This expansion is not limited to consciousness—it goes beyond consciousness, although we will stay within the spectrum of consciousness in this study. This expansion of consciousness conveys a fluid and dynamic change in consciousness as well as a progression through distinct and separate states. The goal of arriving at a state of oneness requires the refinement of the mind beyond thinking to states of feeling, being, and non-being; the refinement of the intellect to intelligence, wisdom, and intuition; and the refinement of the ego to ever-increasing states of insignificance, love, and humility.

In concept, Heartfulness Meditation and Arka Dhyana (and the ancient practices from which derive) focus on a similar endpoint, which is acquired differently and named differently (e.g., pure self, a state of contentless content, union with the absolute, oneness with all). It is noteworthy that the methods to reach that goal differ between the practices.

Changes associated with heart-based meditation practices provide a platform for studying expansion of consciousness from a focus on self to a focus on the universal. Further, these practices form a platform for investigating their resulting impact on daily life (van’t Westeinde and Patel, 2022).

The Awareness Atlas was framed around experience with Heartfulness Meditation. Heartfulness Meditation arose in the early 20th century under the name Sahaj Marg, meaning “the natural path,” and is discussed in detail elsewhere (Patel and Pollock, 2018; van’t Westeinde and Patel, 2022; Patel, 2023). While its roots are in the yogic tradition, it is a new approach, distilling the essence of earlier yogic practices. Heartfulness describes consciousness as the degree of awareness, which also means the degree of unawareness. The Heartfulness practices take a practitioner through changes in mental states and gradually expanding consciousness, and these changes occur through a journey that was originally described by Chandra (1989) and more recently by Patel (2023).

The Awareness Atlas reflects the transition during this journey of expanding consciousness from a focus on self, to a focus that includes others, and eventually that encompasses the oneness of all. This expansion can be understood in terms of increasing awareness and decreasing unawareness.

Pearmain et al. (2023) conducted a phenomenological investigation of the Heartfulness practices by asking participants to describe if and how they noticed the impact of practice as lived experiences in everyday life. Their findings revealed that participants experienced changes in patterns of relating to oneself, others, and the world around them. The findings also identified that participants actively sustained meditative awareness in their hearts. Five core intertwined themes emerged: (1) a shift in focus from thinking to feeling (from head to heart); (2) developing a sense of stability and groundedness (because of being anchored in the heart); (3) being flexible and open, rather than resisting change; (4) sustaining a connection with the heart in the midst of life experiences; (5) immersing oneself in the heart as a space to retreat into, where one is immersed in love and feels empowered.

This phenomenological study provides unique insights into the lived experiences of Heartfulness practitioners and brings into focus ideas of how consciousness might expand in response to a heart-based meditation. However, quantitative methodology is also needed to fully appreciate what expansion of consciousness entails, how it evolves, and the impact on daily life. A combination of quantitative and phenomenological approaches will facilitate exploration of the impact of contemplative practices, such as Heartfulness and others, on expansion of consciousness. Such a combined approach can also be used to explore relationships between changes in consciousness and changes in health, well-being, and development. To this end, it was important to find outcome measures that capture consciousness along the spectrum from a focus on individuality to a focus on oneness with the whole.

### Measures related to consciousness

A number of quantitative measures exist that could be applied to capture changes in the manifestation of consciousness. However, there is a gap: none captures manifestation of consciousness as expressed in daily life through the broad spectrum from individual focus to universal focus. MacDonald and Friedman (2013) reviewed measures from the perspective of transpersonal psychology and presented them in the following categories: spirituality; well-being; experience and consciousness; beliefs, orientation, and identity. Kitson et al. (2020) summarized the psychometric properties of specific characteristics (e.g., gratitude, positive emotions) and for the impact of mindfulness meditation more broadly.

Gamma and Metzinger (2021) developed the Minimal Phenomenal Experience questionnaire (MPE-92), designed to measure the altered states of consciousness experienced by meditators. The MPE-92 has 12 factors that characterize the meditation experience, including time effort and desire; thoughts and feelings; emptiness and non-egoic awareness. A few other scales are available for measuring altered states of consciousness and transcendence specifically related to the effects of psychotropic drugs (Griffiths et al., 2006; Kitson et al., 2020).

Lindhard (2017) developed the Feeling Consciousness Scale based on the impact of the Intuitive Meditation (IM) practice, also referred to as the Arka Dhyana method of meditation. This scale focuses predominantly on identifying changing feelings related to intuition, calmness, and bodily sensations, although a few of the questions relate to the person’s behaviors.

These different measures each focus on a specific aspect of the characteristics in question (e.g., altered states of consciousness, ego, feelings that arise). None of the measures identified by MacDonald and Friedman (2013), nor those identified by Kitson et al. (2020), encompass the spectrum of awareness associated with consciousness as expressed in daily life. The MPE-92 describes the experience of pure awareness as a phenomenon rather than an expression of daily life, and the Feeling of Consciousness scale focuses predominantly on feelings.

### The purpose of this investigation

This investigation was designed to develop a valid measure of the manifestation of consciousness, from the perspective of the Heartfulness practices, that could be applied: (1) to study how shifts in manifestation of consciousness relate to health, well-being, peace, and inclusivity; and (2) to better understand the role of the heart-based practices in expanding manifestation of consciousness.

There is no universally accepted definition of the term consciousness or its manifestations. In this manuscript, we began with Patel’s definition of consciousness as “degree of awareness, which also means degree of unawareness” (van’t Westeinde and Patel, 2022) and adopted an operational definition of manifestation of consciousness as a “state of awareness of self, others, and the connection among all beings.” We took into consideration that consciousness moves along a spectrum, and that expansion of consciousness can result from life experiences that can be viewed as a journey (e.g., normal development, the consequences of life challenges, intentional expansion through activities such as meditation).

## Methods

### Scale development

#### Item generation

In developing this scale, we were cognizant of Western concepts of consciousness, as well as concepts of consciousness from yoga generally (Deshpande, 2021) and the Heartfulness practice specifically. Heartfulness meditation is centered on connection with the heart with an objective of purifying and expanding consciousness. This includes the evolution of thinking to feeling, intelligence to wisdom and intuition, and ego to humility and love (van’t Westeinde and Patel, 2022). Thus, the scale was anticipated to reflect these concepts.

The specific questions included in this measure were based on transcripts from the phenomenological study (Pearmain et al., 2023). Participants in the study were asked the following question: “How does Heartfulness meditation practice affect or permeate your daily life?” The participants then offered their observations without prompting from the interviewer. The transcripts from the phenomenological study were reviewed by one of the investigators of the research team who identified words, concepts, and constructs that could be included in a consciousness scale. Thus, the questions on the Awareness Atlas were derived from words used by people who had practiced Heartfulness Meditation rather than from any preconceived notions of the investigators of this current study. From their review, two members of the research team developed 48 questions for inclusion, including four reverse scored questions. These questions incorporated a broad perspective about expansion of consciousness from the personal and relational items to those that focused on awareness of something beyond oneself. They also specifically reflected the role of the heart in expansion of consciousness. All 48 questions were reviewed, edited, and modified by the entire research team for clarity. All except one member of the team (YJ) practice Heartfulness meditation with experience ranging from 7 to 48 years (average 33 years).

The initial scale consisted of 48 items with 7 themes: (1) Perception of Self; (2) Trust in Self and Others; (3) Relationship to Others; (4) Acceptance of Self; (5) Listening to the Heart; (6) Connection with Higher Self; (7) Acceptance and Letting Go. Four of the items were reverse scored. These items were located in different themes: one reversed question was in Theme 3 Relationship to Others (I strive to get my own way over the needs of others); two were in Theme 4 (It is hard for me to look at my thoughts, actions, and words; I do not feel good about myself); and one reversed question was in Theme 6 (I do not feel connected with a reality that is larger than myself).

#### Content validity

Once the questions had been agreed upon, an iterative process of refining each question was conducted by the researchers. To ensure content and face validity, the questions were piloted through 12 one-on-one interviews to determine how the interviewees understood the questions. We included people who had a meditation practice (Heartfulness [*n* = 3], Buddhist [*n* = 1], Mindfulness [*n* = 4]) or had no meditation practice (*n* = 4). The entire research team then reviewed all suggestions and finalized the wording for the initial 48 questions.

The final survey consisted of an informed consent, followed by demographics variables (e.g., age, sex, education level, geographic location, ethnicity, and meditation practices), and then the 48 questions. The participants were instructed to consider how they actually behave, rather than simply whether they agree or disagree with the statement by reflecting on their experiences over the past 2 weeks. A Likert scale of 1 (never) to 6 (always) was used to rate each statement.

### Testing of the scale

#### Procedure and participants

Institutional review board approval was obtained from the University of Colorado for worldwide distribution with the exception of the European Union (EU). A separate informed consent was approved by the University of Colorado for EU participants. All participants gave informed consent in order to access the survey.

A total of 433 adults aged 18–86 from USA, Canada, India, Europe, and other countries participated in the scale validation process. An invitation was sent to coordinators of Heartfulness Meditation in the participating countries for distribution to meditators in that practice. The invitation invited voluntary participation in an anonymous survey. The invitation was also sent to colleagues, friends, and family members of Heartfulness practitioners, inviting participation from people who participated in other meditation practices, or had no meditation practice.

#### Exploratory and confirmatory factor analysis

Factor analysis is commonly used to establish construct validity. Exploratory factor analysis (EFA) looks at the structure within a large number of items by grouping highly correlated items into factors that reflect different theoretical components of the overall construct (Portney, 2020). After initial screening of the data for missing values and distribution, we conducted an EFA (*n* = 203) using a Principal Axis Factoring as extraction method and promax as rotation method in spss 28. Item screening was based on factor loadings and communalities. Decisions regarding which factors would be retained were made using the following criteria: eigenvalue was greater than 1, inspection of the scree plot, number of items in each factor and factor loadings are all greater than 0.30 on at least one factor, and also taking into account the interpretability of each factor. Internal consistency reliability was assessed using item-scale correlations and the Cronbach alpha (α).

Confirmatory factor analysis (CFA) is used to determine whether the theoretical structure of an instrument fits with current empirical understanding of the construct (Portney, 2020). A second sample of 230 participants was used to conduct CFA in Mplus 7.3. Maximum likelihood with robust error (MLR) estimator was utilized to account for missing and potential non-normal distribution. A second order factor CFA was tested in Mplus as well. We used the most commonly used techniques to evaluate the model fit. This methodology included the chi-square test of exact fit, Root Mean Square Error of Approximation (RMSEA ≤0.06), Tucker Lewis Index (TLI ≥ 0.95), Comparative Fit Index (CFI ≥ 0.95), and Standardized Root Mean Square Residual (SRMR ≤0.08) (Hu and Bentler, 1999). Additionally, the average variance extracted (AVE) was used to assess the convergent validity. If the square root of the AVE of each latent factor is greater than the correlation coefficients between that latent variable and other latent variables in the measurement model, then the model shows evidence of discriminant validity (Fornell and Larcker, 1981).

#### Known-groups validity

Known-groups validity provides further evidence of construct validity, which is provided when the scale can distinguish differences between two or more groups with anticipated differences (Portney, 2020). Known-groups validity of the scale was assessed by examining the association among demographics such as age, sex, type of meditation practice, years of experience of meditation practice and the scale scores. We hypothesized that people with more years of meditation experience would have higher scores on the scale, and other factors would not be highly correlated with scale scores. Further, considering the factors that might affect the scale score collectively vs. independently, a linear regression model with all the above-mentioned factors was entered to explore how those important demographics all together affect the scale score.

#### Test–retest reliability

We used test–retest reliability to examine stability of responses to the scale over time. For this purpose, the final scale was sent to a group of voluntary participants who were asked to respond to the survey twice within a two-week period. The test–retest reliability was calculated using an intraclass correlation coefficient with two-way mixed-effects model and absolute agreement for the total scale score and subscale scores (Koo and Li, 2016).

## Results

### Item reduction and exploratory factor analysis (EFA) results

Data from the first 203 participants were used to conduct the EFA. Participants were 60.6% female, with about half the sample from USA, 24% from Canada, and 26% from India. The mean age was 51.6 (SD = 13.1) and median age was 52.0. Years of meditation ranged from 0 to 50. The mean and median years of meditation were 16.7 (SD = 11.2) and 18, respectively. 83.7% of meditators practiced Heartfulness Meditation, 10.3% had a variety of meditation and spiritual practices, while 5.9% of the sample reported they did not have a meditation practice.

Data screening and cleaning were conducted before running the EFA, including reverse coding for the four reverse wording items. Initial EFA yielded 9 factors with eigenvalue >1; from inspection of the scree plot, a 5-factor solution was determined to be reasonable. Four items were dropped due to very low communality resulting in a five-factor model. The rotated factor structure and loadings revealed that several items had very close double loadings or low loadings (<0.3) at any of the five factors, so those items were dropped.

With the iterative process of the EFA and repetitive evaluation of the factor structure pattern and loadings, and also taking into account interpretability, we arrived at the final 30-item, 5-factor model indicated by the footnote in Table 1. We labeled Factor 1 as Relationship to Self and Others (5 items), Factor 2 as Judgment of Self (4 items), Factor 3 as Listening to the Heart (5 items), Factor 4 as Connection with Higher Self (6 items), Factor 5 as Acceptance and Letting Go (10 items), respectively. These five factors are clearly distinct with very little overlap. The inter-factor correlation coefficients ranged from 0.10 to 0.64 (see Table 2). The five-factor solution with initial eigenvalues ranging from 11.43 to 1.39, explained 61.51% of the total variance.

**Table 1:** EFA results with five factor model (30 items).

Item description	F1	F2	F3	F4	F5
1. I am receptive to the feelings, needs, and suffering of others.	0.546				
2. I notice how others react to me at the time of an interaction.	0.651				
3. I notice my reactions to others at the time of an interaction.	0.543				
4. I consider the feelings, needs, and suffering of others.	0.546				
5. Throughout the day I notice how my thoughts, feelings, and perceptions fluctuate.	0.340				
6. It is hard for me to look at my thoughts, actions, and words.		0.750			
7. I do not feel connected with a reality that is larger than myself.		0.645			
8. I do not feel good about myself.		0.573			
9. I strive to get my own way over the needs of others.		0.471			
10. I listen to the wisdom of my heart (the wisdom that arises when my preconceived notions, desires, judgment, and emotions are silenced) and trust what it tells me.			0.837		
11. I feel guided in life, by the wisdom of my heart.			0.814		
12. When making decisions and interacting with others, it is easy for me to connect with the wisdom of my heart.			0.541		
13. To make decisions in any situation, my heart (inner wisdom) guides me from a place beyond emotion and thought.			0.539		
14. I trust my intuition.			0.588		
15. I feel supported by a deeper reality, underlying all of creation.				0.719	
16. I feel that I am part of something greater than myself.				0.885	
17. I feel a spiritual aspect to my identity, beyond my worldly identity.				0.633	
18. I feel that my consciousness is ever expanding.				0.743	
19. I have a feeling of wonder and awe about life.				0.492	
20. I have a sense of being one with all beings in the universe				0.470	
21. I cheerfully embrace situations that are hard, uncomfortable, or challenging.					0.965
22. I cheerfully adapt to life circumstances in order to grow.					0.913
23. I embrace all experiences of my life with joy as they unfold.					0.694
24. I accept the struggles and lessons in life.					0.756
25. I use my self-awareness to realize I have choices in how to respond to situations.					0.608
26. My emotions, feelings, and thoughts remain balanced (stable) no matter what is going on within and around me.					0.585
27. I consider the perspectives of others and learn from them.					0.555
28. As my awareness and consciousness change, I adapt my behaviors in order to be compatible with these changes.					0.527
29. I trust that all will work out as is necessary, even in the most difficult situations.					0.531
30. I have a feeling of gratitude no matter what I encounter throughout the day.					0.516
					
Initial Eigenvalues	1.513	1.394	1.816	2.302	11.428
% variance explained	5.045	4.647	6.052	7.674	38.092

Factor loadings < 0.3 was not showing in the table; items in factor 2 were reverse coded. F1 = Relationship to Self and Others (5 items), F2 = Judgment of Self (4 Items), F3 = Listening to the Heart (5 items), F4 = Connection with Higher Self (6 items), F5 = Acceptance and Letting Go (10 items).

**Table 2:** Inter-factor correlations with EFA five factor model factor correlation matrix.

Factor	1	2	3	4	5
1. Relationship to Self and Others	1.000				
2. Judgment of Self	0.605	1.000			
3. Listening to the Heart	0.644	0.566	1.000		
4. Connection with Higher Self	0.574	0.428	0.470	1.000	
5. Acceptance and Letting Go	0.098	0.185	0.141	0.215	1.000

Extraction Method: Principal Axis Factoring. Rotation Method: Promax with Kaiser Normalization.

### Confirmatory factor analysis (CFA) results

A second sample of 230 participants was used for the CFA. Participants were 68.7% female, with 56% of the sample from Europe, 25% from USA, 13% from India, 4.3% from Canada, and 1.7% from other countries. The mean age was 54.2 (SD = 13.4) and median age was 55.5. Years of meditation ranged from 0 to 58. The mean and median years of meditation were 18.1 (SD = 12.1) and 19, respectively. 78.9% of meditators practiced Heartfulness Meditation, 16% had a variety of meditation and spiritual practices, while 5.2% did not have a meditation practice.

A five-factor model was specified initially, and items 5, 6 and 9 had factor loadings <0.30, so all three items were dropped from the CFA analysis. These two dropped items were originally from the factor Judgment of Self in the EFA five-factor model. After dropping two items in the factor Judgment of Self, only items 7 and 8 remained. Following a common practice with a minimum of three items in each factor, a four-factor CFA model was also specified. Moreover, modification indices indicated items 29 and 30 could potentially load on another factor—Connection with Higher Self. An iteration of model comparisons was carried out among four-factor and five-factor models, as well as keeping or dropping items 7 and 8, or moving 29 and 30 to a different factor, resulting in the best model with a total of 23 items with four factors: Relationship to Others (RO, 5 items), Listening to the Heart (LH, 5 items), Connection with Higher Self (CHS, 6 items), Acceptance and Letting Go (ALG, 7 items). See the final scale and factor loadings in Table 3. The final scale with instructions is available in the Supplementary material.

**Table 3:** CFA results with four factor model (23 items).

	Final item number	Loadings	Alpha	Test–retest reliability	Composite reliability	AVE
**Relationship to Others** (5 items)		**0.691#**	0.814	0.713	0.817	0.473
1. I am receptive to the feelings, needs, and suffering of others.	RO1	0.767				
2. I notice how others react to me at the time of an interaction.	RO2	0.663				
3. I notice my reactions to others at the time of an interaction.	RO3	0.656				
4. I consider the feelings, needs, and suffering of others.	RO4	0.749				
27. I consider the perspectives of others and learn from them.	RO5	0.626				
**Listening to the Heart** (5 items)		**0.937#**	0.919	0.911	0.918	0.694
10. I listen to the wisdom of my heart (the wisdom that arises when my)	LH1	0.823				
Preconceived notions, desires, judgment, and emotions are silenced and trust what it tells me.						
11. I feel guided in life, by the wisdom of my heart.	LH2	0.872				
12. When making decisions and interacting with others, it is easy for me to connect with the wisdom of my heart.	LH3	0.856				
13. To make decisions in any situation, my heart (inner wisdom) guides me from a place beyond emotion and thought.	LH4	0.869				
14. I trust my intuition.	LH5	0.713				
**Connection with Higher Self** (6 items)		**0.821#**	0.890	0.920	0.885	0.568
15. I feel supported by a deeper reality, underlying all of creation.	CHS1	0.841				
16. I feel that I am part of something greater than myself.	CHS2	0.741				
17. I feel a spiritual aspect to my identity, beyond my worldly identity.	CHS3	0.765				
18. I feel that my consciousness is ever expanding.	CHS4	0.764				
19. I have a feeling of wonder and awe about life.	CHS5	0.609				
20. I have a sense of being one with all beings in the universe.	CHS6	0.744				
**Acceptance and Letting Go** (7 items)		**0.878#**	0.897	0.762	0.898	0.562
21. I cheerfully embrace situations that are hard, uncomfortable, or challenging.	ALG1	0.776				
22. I cheerfully adapt to life circumstances in order to grow.	ALG2	0.817				
23. I embrace all experiences of my life with joy as they unfold.	ALG3	0.838				
24. I accept the struggles and lessons in life.	ALG4	0.620				
25. I use my self-awareness to realize I have choices in how to respond to situations.	ALG5	0.674				
26. My emotions, feelings, and thoughts remain balanced (stable) no matter what is going on within and around me.	ALG6	0.746				
28. As my awareness and consciousness change, I adapt my behaviors in order to be compatible with these changes.	ALG7	0.708				
Overall scale			0.950	0.900	0.900	0.700

# Bold indicates the second-order factor loadings. AVE, Average variance extracted.

Of note, the four reverse scored items were designed to belong to different themes at the early stage of the scale. However, the EFA results indicated that all the four items converged to one distinct factor, which we labeled Judgment of Self and which was dropped as described above.

**Figure 1:**
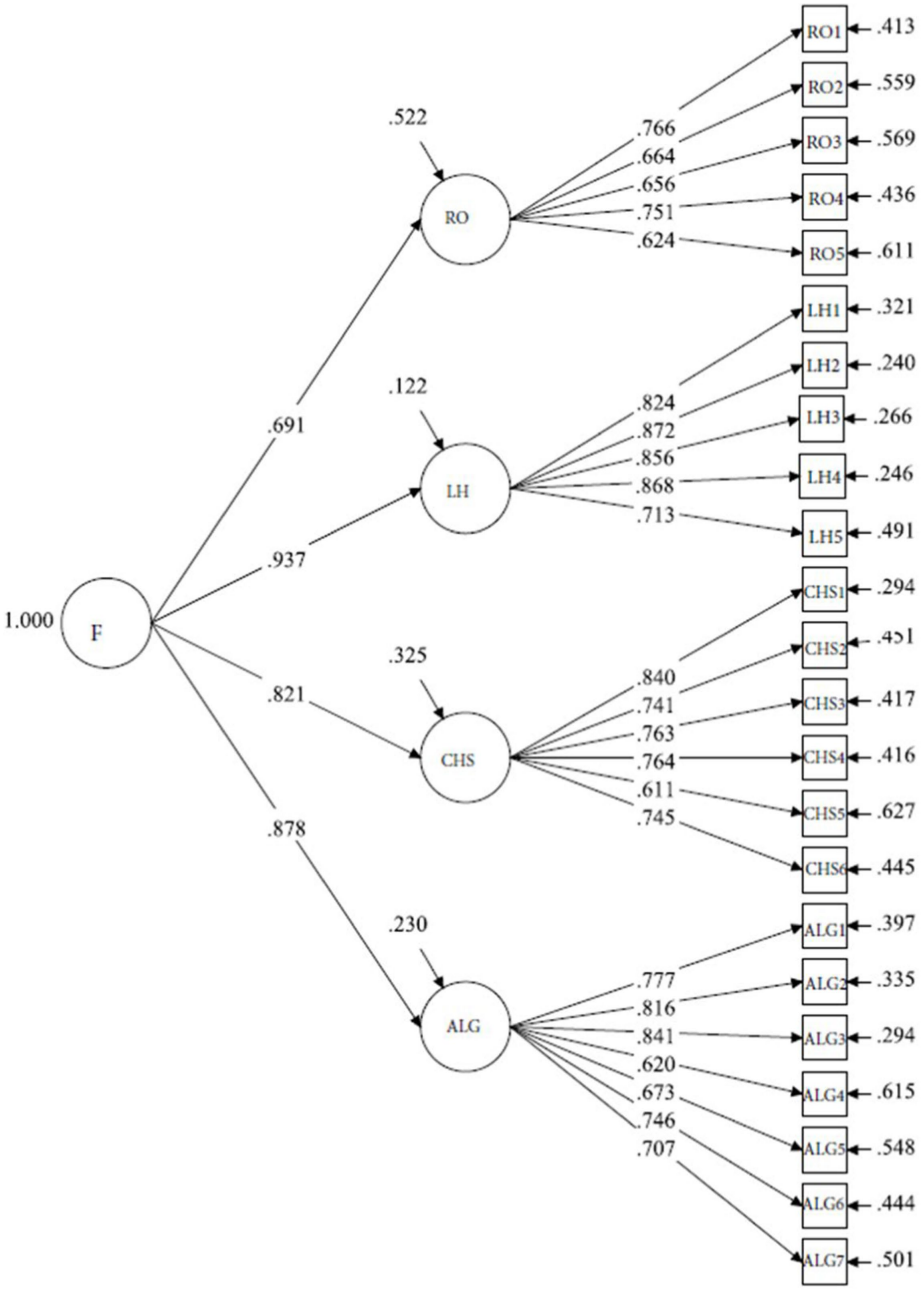
Graph representation of the CFA of the second order factor model. The second order factor CFA model fit indices (Chi-square = 356.60, df = 223, RMSEA = 0.051, CFI = 0.95, TLI = 0.94, SRMR = 0.048) was excellent. All 23 items loaded as we originally conceptualized, with all loadings greater than 0.60. Two pairs of residual correlations (CHS1 & CHS2, ALG1 & ALG2) were added to improve the model fit.

The latent factor correlations in the final four-factor model were relatively high, ranging from 0.56 to 0.82, hence a second-order factor model was specified in Mplus to investigate if a general construct exists. The chi-square difference test (=0.65) between the first order four-factor model and the second order factor model did not show significant difference, providing evidence of retaining the second-order factor model. A graph representation of the second-order factor model can be seen in Figure 1.

Confirmatory factor analysis confirmed four factors for the final scale with excellent model fit indices (Chi-square = 355.95, df = 221, RMSEA = 0.052, CFI = 0.95, TLI = 0.94, SRMR = 0.048); similarly, the second-order factor model showed favorable model fit indices as well (Chi-square = 356.60, df = 223, RMSEA = 0.051, CFI = 0.95, TLI = 0.94, SRMR = 0.048). All 23 items loaded as we originally conceptualized, with all loadings greater than 0.60 as presented in Table 3. The final four-factor model with 23 items has excellent overall internal consistency reliability (alpha = 0.95), each factor’s consistency reliability ranging from 0.81 to 0.91, each factor’s composite reliability ranging from 0.82 to 0.92 (Table 3), indicating excellent internal and composite reliability. This final scale had a mean score of 108.63 ± 16.20 out of a possible 138, with an observed range from 59 to 138. Item-scale correlations ranged from 0.52 to 0.82.

Construct validity was demonstrated by both EFA and CFA, both first order four-factor model and second order factor model showed a favorable model fit and meaningful factor loadings. The second order factor model provided support to the underlying latent construct which we termed “manifestation of consciousness” as a general construct. The subscales were highly correlated with the total scale score ranging from 0.73 to 0.89, but moderately correlated with each other, indicating good construct validity. The average variance extracted (AVE) for each factor was greater than 0.5 (Table 3), except for the first factor, Relationship to Others, which had an AVE of 0.47, indicating good convergent validity overall. Although the AVE of this specific factor is just below 0.5, this factor’s composite reliability is above 0.70; hence we deemed this factor acceptable (Fornell and Larcker, 1981). Moreover, the square root of AVE for all factors is greater than their correlations with other factors, indicating good discriminant validity.

### Known-groups validity

**Figure 2:**
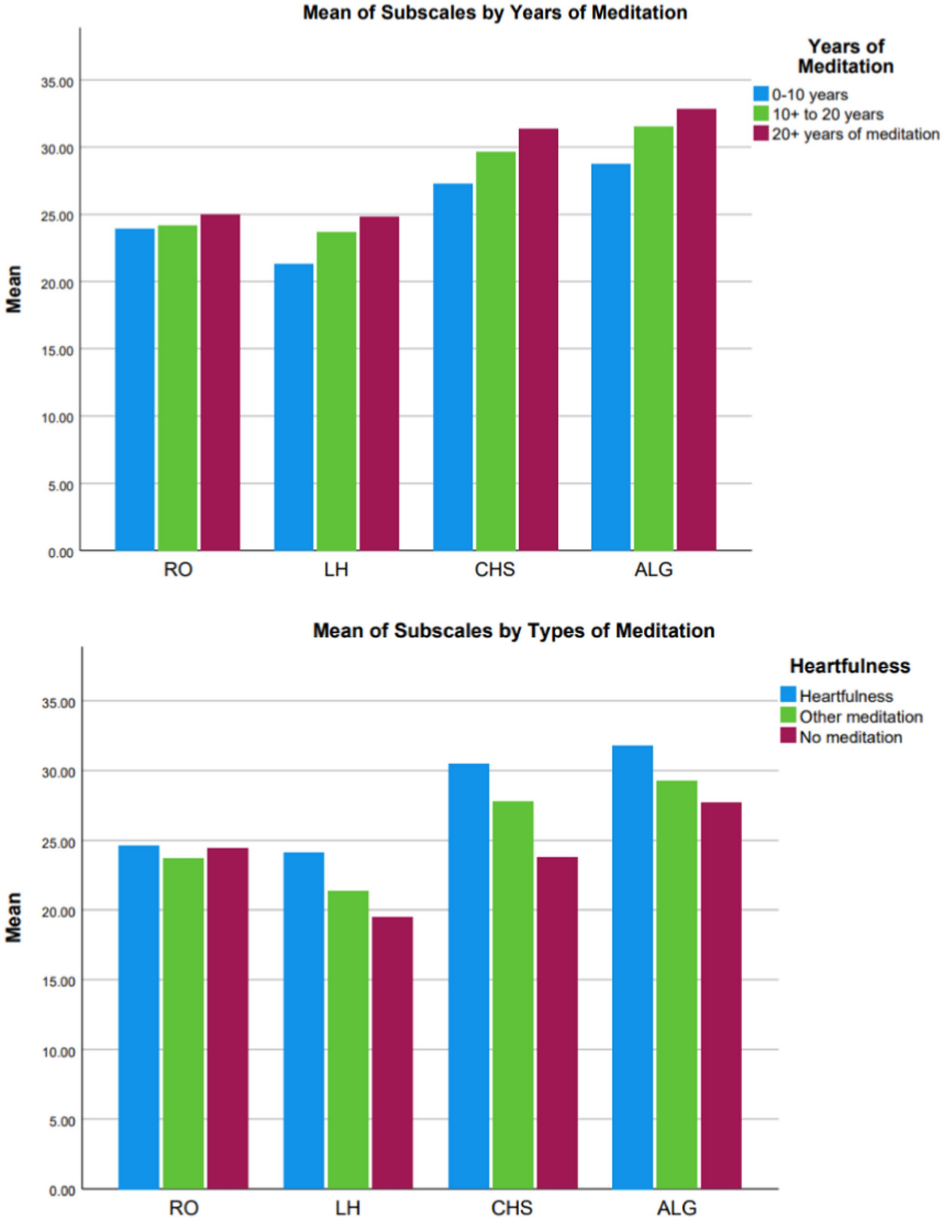
Comparison subscale means by years of meditation and types of meditation. RO, Relationship with Others; LH, Listening to the Heart; CHS, Connection with Higher Self; ALG, Acceptance and Letting Go. The subscales of LH, CHS, ALG showed significantly different among <= 10, 10–20, and > 20 years of meditation, while only 20+ years of meditators showed significant higher scores on subscale RO than <=10 and 10–20 years, and no difference was found between <=10 and 10–20 years of meditation. Similarly, heartfulness meditators showed significantly higher scores than other meditators and other meditators showed significantly higher score than no meditation on LH, CHS, ALG score, while no difference was found on RO score.

The known-groups validity of the final scale was examined by comparing the scale scores by demographic groups (Table 4). In particular, participants’ years of meditation were found to be significantly correlated with all subscale scores and total score (*p* < 0.05) (Figure 2). Furthermore, participants with more years of meditation scored higher on all subscales scores and the total score. Males and females did not show significant differences on the total score and three of the subscale scores. However, females scored higher in the factor Connection with Higher Self than males (*p* = 0.008). Additionally, age was not correlated with two subscales, and had small correlations (Rs < = 0.15, *p* < 0.05) with subscale of LH and CHS as well as the total score. Participants who practice Heartfulness meditation scored significantly higher than other meditation and no meditation on the total scale and subscale scores, with one exception of subscale Relationship to Others. Finally, participants from Asian descents were found to score higher in the total scale score (Asian = 110.61 vs. non-Asian = 105.73, *p* = 0.01) and the subscales of Listening to the Heart (p = 0.01) and Acceptance and Letting Go (*p* = 0.005). Finally, we used a regression model with age, sex, years of meditation, Heartfulness vs. other, and Asian vs. non-Asian as predictors to predict total scale score. The results indicated that only years of meditation and practicing Heartfulness mediation were significantly associated with the total scale score. Whether respondents were of Asian descent no longer significantly associated with the total score.

**Table 4:** Known-groups validity evidence with the combined sample (*n* = 449).

	*N*	RO	LH	CHS	ALG	Total score
**Age**	430	0.059	0.104*	0.154**	0.075	0.124*
**Years Med**	426	0.107*	0.293**	0.303**	0.272**	0.308**
**Sex**		M ± SD	M ± SD	M ± SD	M ± SD	M ± SD
Female	281	24.61 ± 3.20	23.49 ± 4.39	30.13 ± 5.34	31.01 ± 5.94	109.17 ± 16.00
Male	149	24.17 ± 3.21	23.17 ± 4.95	28.68 ± 5.44	31.40 ± 5.89	107.52 ± 16.62
		–	–	***p* = 0.008**	–	–
**Years Med**						
< = 5 Years	103	23.92 ± 3.20	21.20 ± 4.70	27.04 ± 6.59	28.57 ± 5.92	100.56 ± 16.78
> 5 Years	323	24.64 ± 3.17	24.13 ± 4.22	30.50 ± 4.59	32.00 ± 5.65	111.26 ± 14.90
		***p* = 0.047**	***p* < 0.001**	***p* < 0.001**	***p* < 0.001**	***p* < 0.001**
0–10 Years	145	24.12 ± 3.21	21.65 ± 4.73	27.71 ± 6.43	28.97 ± 5.78	102.36 ± 16.63
11–20 Years	120	24.15 ± 3.14	23.65 ± 4.30	29.65 ± 4.86	31.55 ± 5.57	109.00 ± 15.42
20+ Years	161	25.01 ± 3.14	24.85 ± 3.92	31.43 ± 3.84	32.86 ± 5.63	114.16 ± 13.78
		***p* = 0.021**	***p* < 0.001**	***p* < 0.001**	***p* < 0.001**	***p* < 0.001**
**Ethnicity**						
Asian	194	24.61 ± 3.18	24.03 ± 4.49	29.96 ± 5.29	32.01 ± 6.01	110.61 ± 16.46
Non-Asian	101	24.57 ± 2.75	22.56 ± 4.74	28.71 ± 6.28	30.01 ± 5.25	105.73 ± 14.92
		–	***p* = 0.010**	–	***p* = 0.005**	***p* = 0.013**
**Heartfulness**						
Heartfulness	338	24.64 ± 2.91	24.09 ± 4.38	30.46 ± 4.79	31.82 ± 5.86	111.00 ± 15.53
Other	94	23.97 ± 3.20	24.70 ± 4.61	26.51 ± 6.45	28.80 ± 5.21	99.80 ± 15.07
		–	***p* < 0.001**	***p* < 0.001**	***p* < 0.001**	***p* < 0.001**
All	449	24.40 ± 3.20	23.23 ± 4.69	29.41 ± 5.57	31.03 ± 5.93	108.06 ± 16.46

*p < 0.05, **p < 0.01. RO, Relationship to Others; LH, Listening to the Heart; CHS, Connection with Higher Self; ALG, Acceptance and Letting Go. – indicates *p* > 0.05.

### Test–retest reliability

Thirty-six participants took the survey twice within a two-week period (average time period between the test and retest was 13.7 days). The test–retest reliability (i.e., intraclass correlation coefficient) for the total score was 0.90, and subscale scores test–retest reliability ranged from 0.71 to 0.92.

## Discussion

This manuscript describes the development and testing of a scale, known as the Awareness Atlas, which measures the manifestation of consciousness in daily life. The final model fit exceeded all the required thresholds, indicating an excellent fitted model with a single dimensionality to measure the manifestation of consciousness with four domains: Relationship to Others (RO), Listening to the Heart (LH), Connection with Higher Self (CHS), and Acceptance and Letting Go (ALG). Additionally, the final scale explained more than 50% of the total variance and all the factor loadings were greater than the recommended threshold of 0.4 (Boateng et al., 2018). Together, our findings indicate a measurement model for everyday aspects of manifestation of consciousness.

This manuscript also offers new insights into the current discussion of consciousness. We considered a wide range of definitions drawing from Eastern and Western perspectives, philosophical and modern scientific perspectives, experts and everyday people who experience shifts in consciousness in daily life. We then used this background to support us in developing an operational definition with an aim to develop a simple, comprehensive, and globally-relevant tool.

### Construction of the scale

To develop the scale, we began with concepts of consciousness from Heartfulness Meditation, a set of simple practices based on the philosophy underlying Raja Yoga meditation (Chandra, 1989; van’t Westeinde and Patel, 2022; Patel, 2023). The scale emphasizes a connection with the heart. The questions included in the scale were informed by transcripts from participants in a phenomenological study of the lived experience of Heartfulness (Pearmain et al., 2023). The Awareness Atlas was developed from responses of participants in the preliminary phenomenological study. Further, the four factors were derived by statistical analysis from the data.

With the intention of covering as many aspects of lived experiences narrated by respondents as possible, the initial scale started off with an item pool of 48. The number was gradually reduced by following specific criteria at different steps of the factor analysis models, as detailed in the results section. The final scale with 23 items was comprehensively representative of our definition of manifestation of consciousness, which is evident from the final factor structure, factor loadings (all >0.6), and total variance explained (>50%) (Figure 1). The emerging four domains of the new Awareness Atlas represent distinct areas of contribution to the overall manifestation of consciousness. These domains are Relationship to Others (5 items), Listening to the Heart (5 items), Connection with Higher Self (6 items), and Acceptance and Letting Go (7 items). Each of these content areas has counterparts in the focus of scales developed by others, e.g., the Feelings, Reactions, Beliefs Summary (Cartwright and Mori, 1988); Ego Grasping Orientation (Knoblauch and Falconer, 1986); Self-Expansiveness level Form (Friedman, 1983), however, no scale that we found encompassed all areas in a single scale.

### Psychometric properties

By following all the necessary steps of scale development (Boateng et al., 2018), the newly developed scale demonstrated excellent validity and reliability in measuring our definition of the manifestation of consciousness. Specifically, in the confirmatory factor analysis testing dimensionality, the final second-order factor model fit exceeded all the required thresholds, indicating an excellent fitted model to measure a general construct—the manifestation of consciousness—with four domains.

Although all four factors demostrated acceptable to excellent test–retest reliability, it is noteworthy that Factor 1 (Relationships to Others) had a relatively lower test–retest reliability (0.71) and higher residuals for the items than the other factors. Nevertheless, the reliability and loadings exceeded the recommended thresholds. This finding, which should be further examined in future studies, might reflect the day-to-day fluctuation in a person’s relationship to others. It is possible that life stresses or a particularly relaxed period of time might have influenced how the respondents rated their relationships to others more than other factors, which might be more stable over time.

The Awareness Atlas compares favorably in its psychometric properties with existing tools. For example, the Intellectual Humility Scale (Krumrei-Mancuso and Rouse, 2016) had comparable psychometric properties (internal consistency coefficient *α* = 0.82 to 0.88, with subscales at 0.70 to 0.89 across samples); the MPE-92 (Gamma and Metzinger, 2021) reported internal reliability coefficients for 12 factors between 0.52 and 0.82; the State Mindfulness Scale (Tanay and Bernstein, 2013) with sound internal consistency where the total score coefficient *α* = 0.95 with two subscales 0.90 and 0.95 but small to moderate test–retest reliability, with control condition ranging 0.46 to 0.59 and intervention condition ranging 0.22 to 0.68 with a two-week period. In contrast, both internal consistency reliability and test–retest reliability of our final scale favorably meet the criteria for scale development, as the overall scale reliability was greater than 0.90, and all subscale reliability scores were above 0.70. Similarly, the CFA fit indices (CFI & TLI > =0.94, SRMR & RMSEA around 0.05) of our final scale were excellently comparable to the abovementioned scales, while the existing related scales with acceptable fit where CFI & TLI around 0.90 and RMSEA & SRMR around 0.08.

Of note, the Feeling Consciousness Scale (FCS), developed by Lindhard (2017, 2018a), focuses exclusively on the third level of consciousness, i.e., feeling-mind, one of the six levels outlined in Arka’s theory (Lindhard, 2017). In their study, the FCS was employed to assess pre and post training effects of intuitive meditation conducted over a 6-week period. The author has acknowledged the need for further refinement and validation of the scale, due to a small sample size (*n* = 31) and limited evidence of validity and reliability.

We also examined known-groups validity by examining how the scale scores related to important demographics subgroups. Age and sex were not related to the total scale score, while participants who had more years of meditation were found to have significantly higher scores for all subscale scores and the total score than those who has fewer years of meditation, indicating good construct validity. Of note, those practicing Heartfulness meditation scored significantly higher than other meditation and no meditation groups. Given that the scale was constructed using Heartfulness meditation (a practice focused on heart-based awareness) as a basis, these findings add further support for known-groups validity of the Awareness Atlas.

In summary, our findings indicated a well-constructed instrument for measuring the manifestation of consciousness. The final scale has very strong fit indices and psychometric properties, consists of a short number of items, yet is sufficient to measure the desired construct. This new scale differs from existing scales related to consciousness (Mylonas et al., 2012; Gamma and Metzinger, 2021), in that it focuses on how consciousness manifests in relation to the experience of daily life. This new scale positions us well for studies examining how consciousness manifests in individuals whether or not they have experience with meditation.

### Factors affecting scale scores

We conducted exploratory analyses of the data in this initial study to further understand the scale and to guide future research. Given that meditation practices originated in the East and are generally well-accepted by people of Asian descent, we examined scores of people of Asian descent compared to those who were not of Asian descent. We also examined the relationship between years of meditation and both total and subscale scores. Participants who had a longer history of meditation scored higher on the scale scores, those of Asian descent scored higher than those of non-Asian descent on the total score, and participants who practiced Heartfulness meditation scored significantly higher than other meditation and no mediation groups. Given the difference in numbers of participants in each of these groups, and the fact that the participants in “other meditations” and “no meditation” were recruited by Heartfulness practitioners, we acknowledge the need for a future study with more balanced sample sizes in each group. When we examined a number of factors in a multivariate analysis (i.e., age, sex, years of meditation, Heartfulness vs. other, Asian vs. non-Asian) to predict the total scale score, we found that only years of meditation generally, and Heartfulness meditation specifically, significantly predicted the total scale score. It is noteworthy that age was not a significant contributor in the regression analysis, indicating that it was not a factor of importance in this sample. These findings lend support to the good validity of the scale, suggesting it can be used with people having a wide range of backgrounds and ages.

An outcome of Heartfulness meditation is expanded consciousness (van’t Westeinde and Patel, 2022). The finding that those who meditated for more years had higher scores than those who meditated for fewer years (Table 4; Figure 2) suggests that meditation does indeed alter manifestation of consciousness. Further, future longitudinal studies are needed to determine the extent to which evolution occurs with respect to feeling, wisdom and intuition, and humility with a meditation practice focused on expanding consciousness. Furthermore, future studies are needed to determine whether specific aspects of expanding consciousness are affected differently depending on the nature and purpose of the person’s meditation practice.

Findings from this study demonstrate that the Awareness Atlas provides a tool that can further explore how reliance on the heart impacts a person’s inner sense and feelings. Specifically, findings from the previous phenomenological study (Pearmain et al., 2023) suggest that participants found a sense of security, a groundedness within themselves, and a feeling of being held within something larger than self. They also identified a heart space that was very important to them. Those participants described improved relationships with themselves and with others. Taken together, these findings suggest there may be a growing capacity to relate with oneself and with others as a feeling of security increases. This is an interesting observation when viewed from the perspective of the innate need to feel that one has a place of belonging within and in relationship to others (Ainsworth, 1963; Bowlby, 1973). While we acknowledge the limitations in the current study, we believe that the Awareness Atlas can be used to explore such relationships in future studies including further exploration of the concept of a “heart space” to fully appreciate this new dimension to the understanding of health, wellness, and consciousness. Such investigations should explore the relationship between the heart space, the feeling of security, and people’s ability to relate and manage life circumstances differently (e.g., relationship with self and others, the ability to navigate through difficult circumstances). Combined use of the Awareness Atlas with a phenomenological approach may help to elucidate new and important areas of investigation. Additionally, it will be important to further examine whether this scale has similar performance characteristics in other heart-based practices. Such studies could lead to a new understanding of the role of heart awareness and expansion of consciousness beyond conscious cognitive awareness.

Possibly, when one feels more grounded and secure within oneself it is easier to be more open and receptive to others, and to better navigate life’s difficulties, including health challenges. A body of literature has examined the role of religion and spirituality in health with findings suggesting a positive association between meditation, health behaviors, and subjective well-being (e.g., Jayanna, 2023). Evidence is still needed regarding causality (Bożek et al., 2020; Ransome, 2020). George et al. (2000) considered possible reasons why spirituality and religion might contribute to health, including the encouragement of health promotion by religions, as well as social support that comes with religious and spiritual practices. These authors suggested that a third possibility was most compelling to them, namely the possibility that religion provides a sense of coherence and meaning, so that people develop a better understanding of their place in the universe, their purpose in life, and are better able to endure difficulties when they occur. Ransome (2020) has called for new tools that would be appropriate to epidemiological studies to further understand these relationships in a broader context. The Awareness Atlas provides such an option.

The Awareness Atlas is also applicable to investigations beyond religious and spiritual practices. For example, this scale can be used to explore how consciousness relates to the emotional impact of acute and chronic conditions. It is worth exploring whether reliance on the wisdom of the heart and having an expanded consciousness contributes to why certain people manage disease and aging more easily than others. Further, if expanded consciousness is positively associated with successfully navigating chronic conditions, there may be opportunities to assist people living with such conditions to improve their quality of life through approaches that expand consciousness. These and related issues are worthy of scientific exploration, utilizing both quantitative and qualitative approaches.

There is a growing awareness of the importance of heart rate variability (HRV) in creating emotional stability and a sense of inner calm (McCraty and Zayas, 2014; McCraty et al., 2017). Specifically, heart rate variability provides a measure of physiological coherence. Social coherence has been described as the organization or regulation of groups of individuals that are connected. It has been proposed that individual coherence (measured through HRV) and collective heart rate coherence can be used to increase group coherence (McCraty et al., 2017).

Finally, the operational definition used in this manuscript, “state of awareness of self, others, and the connection among all beings” encompasses the idea that consciousness is a dynamic state of mind, akin to awareness, which expands as one grows and evolves. Various schools of yoga and meditation aim to expand this awareness so that individuals begin to think more broadly of other members in the society, other life forms, the planet, and the universe. This idea appears to be more relevant today when societies are faced with challenges such as wars and terrorism, self-centered economy and development, growing inequities between regions, global warming, etc. Expansion of consciousness appears critical to improving these difficult societal and personal problems.

There are limitations to this study. Participants enrolled in the study were self-selected, which may introduce a potential selection bias. Most of the participants had practiced some form of meditation and many practiced Heartfulness meditation specifically. Additionally, those who meditated in other practices or did not have a meditation practice were recruited through friends and family members of Heartfulness meditators. We are aware of the biases inherent in this approach and will use an alternate recruitment method in further studies.

In this first investigation, we explored only a few factors for their contributions to responses (e.g., years of meditation, sex). A robust examination of potential contributors to scores on the Awareness Atlas should be undertaken in the future including a more comprehensive examination of the role of geographic location and the role of ethnicity in score responses. In this first investigation we did not examine concurrent validity by assessing the association of response on the newly developed scale with other existing scales. This should be evaluated in future studies to further establish the validity of the scale.

Additionally, the final scale does not include reverse scored items. Although the four reverse scored items were designed to belong to different themes at the early stage of the scale, the EFA results indicated that all the four items formed one distinct factor which we labeled ‘Judgment of Self.’ With an iterative comparison of the model fit, the final scale dropped all the reversed items resulting in a four-factor model. Future investigations should examine why the reverse scored items performed very differently than their counterparts of positively scored items.

It should be noted that the EFA sample did not include European participants, whereas the CFA incorporated participants from Europe. Despite this distinction, the EFA and CFA samples exhibited similarities in sex, age, educational level, and average years of meditation practices. Further validation among culturally diverse groups via measurement invariance will be the next step for future validations.

Despite the limitations above, our study managed to attract a diverse range of participants from various continents. This diversity and the size of the overall sample enhances the generalizability of the findings.

## Conclusion

The Awareness Atlas was developed to measure the manifestation of consciousness through the lens of lived experience. This new measure quantifies manifestation of consciousness across a spectrum from simple awareness of self and others to an awareness of that which is beyond self. The Awareness Atlas has strong psychometric properties. It positions scientists to explore questions about changes in the manifestation of consciousness with practices designed to expand awareness (e.g., yoga, meditation) and with life experiences that affect awareness (e.g., life-altering conditions), as well as in response of communities to global challenges (e.g., climate change, wars, famines). Finally, the newly developed Awareness Atlas also can be used by individuals for self-reflection.

## Data availability statement

The raw data supporting the conclusions of this article will be made available by the authors, without undue reservation.

## Ethics statement

Institutional review board approval was obtained from the University of Colorado for worldwide distribution with the exception of the European Union (EU). A separate informed consent was approved and obtained from the University of Colorado for EU participants. All participants gave informed consent in order to access the survey.

## Author contributions

YJ: Data curation, Formal analysis, Methodology, Project administration, Validation, Writing – original draft, Writing – review & editing. MS: Conceptualization, Data curation, Investigation, Project administration, Supervision, Validation, Writing – original draft, Writing – review & editing. HC: Conceptualization, Investigation, Writing – review & editing. KJ: Conceptualization, Investigation, Writing – review & editing. RP: Conceptualization, Investigation, Writing – review & editing. AV: Writing – review & editing, Conceptualization, Investigation. KP: Writing – review & editing, Conceptualization, Investigation.

## References

[ref1] Ainsworth M. D. S. (1963). “The development of infant-mother interaction among the Ganda” in Determinants of Infant Behavior. ed. Foss B. M. (London: Methuen), 57–112.

[ref3] Boateng G. O. Neilands T. B. Frongillo E. A. Melgar-Quiñonez H. R. Young S. L. (2018). Best practices for developing and validating scales for health, social, and behavioral research: a primer. Front. Public Health 6:149. doi: 10.3389/fpubh.2018.00149 29942800 PMC6004510

[ref4] Bowlby J. (1973). Attachment and Loss. Vol 11. Separation: Anxiety and Anger. New York: Basic Books.

[ref5] Bożek A. Nowak P. F. Blukacz M. (2020). The relationship between spirituality, health-related behavior, and psychological well-being. Front. Psychol. 11:1997. doi: 10.3389/fpsyg.2020.01997 32922340 PMC7457021

[ref6] Cartwright D. Mori C. (1988). Scales for assessing aspects of the person. Person Cent. Rev. 3, 176–194.

[ref7] Carver C. S. Glass D. C. (1976). The Self-Consciousness Scale: A Discriminant Validity Study. J. Pers. Assess. 40, 169–172. doi: 10.1207/s15327752jpa4002_8, PMID: 16367389

[ref8] Chandra R. (1989). Complete Works of Ram Chandra, Volume 1. Toronto, CA: SRCM.

[ref9] Deshpande P.Y. (2021). The Authentic Yoga—Yoga Sutras of Patanjali. 2nd. Pragati Offset Pvt Ltd: Hyderabad, India.

[ref10] Diemer M. A. Rapa L. J. Park C. J. Perry J. C. (2017). Development and Validation of the Critical Consciousness Scale. Youth Soc. 49, 461–483. doi: 10.1177/0044118X14538289, PMID: 37729117

[ref12] Fornell C. D. Larcker D. F. (1981). Evaluating Structural Equation Models with Unobservable Variables and Measurement Error. J. Mark. Res. 18, 39–50. doi: 10.1177/002224378101800104, PMID: 33691717

[ref13] Friedman H. L. (1983). The self-expansive level form: A conceptualization and measurement of a transpersonal construct. J. Transpers. Psychol. 15, 37–50.

[ref14] Gamma A. Metzinger T. (2021). The Minimal Phenomenal Experience Questionnaire (MPE-92M): Towards a phenomenological profile of “pure awareness” experiences in meditators. PLoS One 16:e0253694. doi: 10.1371/journal.pone.0253694, PMID: 34260614 PMC8279394

[ref15] Garcia-Castro J. Kodukula P. (2022). Why we need a whole new approach into the study of consciousness. J. Conscious. Exp. Res. 13, 397–414.

[ref16] George L. K. Larson D. B. Koenig H. G. McCullough M. E. (2000). Spirituality and health: What we know, what we need to know. J. Soc. Clin. Psychol. 19, 102–116. doi: 10.1521/jscp.2000.19.1.102, PMID: 38031456

[ref17] Grant A. M. Franklin J. Langford P. (2002). The self-reflection and insight scale: a new measure of private self-consciousness. Soc. Behav. Personal. Int. J. 30, 821–835. doi: 10.2224/sbp.2002.30.8.821

[ref18] Griffiths R. R. Richards W. A. McCann U. Jesse R. (2006). Psilocybin can occasion mystical-type experiences having substantial and sustained personal meaning and spiritual significance. Psychopharmacology 187, 268–283. doi: 10.1007/s00213-006-0457-5, PMID: 16826400

[ref19] Guertin P. A. (2019). A novel concept introducing the idea of continuously changing levels of consciousness. J. Conscious. Explor. Res. 10, 406–412.

[ref20] Hu L. T. Bentler P. M. (1999). Cutoff criteria for fit indexes in covariance structure analysis: conventional criteria versus new alternatives. Struct. Equ. Model. Multidiscip. J. 6, 1–55. doi: 10.1080/10705519909540118, PMID: 36787513

[ref21] Jayanna K. (2021). Meditation and Expanded Consciousness: Implications for Sustainable Health and Wellbeing. Consciousness and Meditation: Benefits, Applications and Research: Bangalore, India.

[ref22] Jayanna K. (2023). Integrative approach to lifestyle management: implications for public health research & practice in the context of SDG-3. J. Ayurveda Integr. Med. 14:100796. doi: 10.1016/j.jaim.2023.100796, PMID: 37738855 PMC10692374

[ref23] Kegan R. (1982). The Evolving Self. Harvard University Press, Cambridge, MA.

[ref24] Kihlstrom J. F. (2022). Consciousness, the unconscious, and the self. Psychol. Conscious. Theory Res. Pract. 9, 78–92. doi: 10.1037/cns0000285

[ref25] Kirsmayer L. J. (2010). Peace, conflict and reconciliations: contributions of cultural psychiatry. Transcult. Psychiatry 47, 5–19. doi: 10.1177/1363461510362037, PMID: 20511248

[ref26] Kitson A. Chirico A. Gaggioli A. Riecke B. E. (2020). A review on research and evaluation methods for investigating self-transcendence. Front. Psychol. 11:547687. doi: 10.3389/fpsyg.2020.547687 33312147 PMC7701337

[ref27] Knoblauch D. L. Falconer J. A. (1986). The relationship of a measured Taoist orientation to Western personality dimensions. J. Transpers. Psychol. 18, 73–83.

[ref28] Koo T. K. Li M. Y. (2016). A guideline of selecting and reporting intraclass correlation coefficients for reliability research. J. Chiropr. Med. 15, 155–163. doi: 10.1016/j.jcm.2016.02.012, PMID: 27330520 PMC4913118

[ref29] Krumrei-Mancuso E. J. Rouse S. V. (2016). The development and validation of the comprehensive intellectual humility scale. J. Pers. Assess. 98, 209–221. doi: 10.1080/00223891.2015.1068174 26542408

[ref30] Lindhard T. (2017). Experiencing peace through heart-based meditation on the Self. Open Psychol. J. 10, 27–40. doi: 10.2174/1874350101710010027

[ref31] Lindhard T. (2018a). The Theory of Six Main Levels of Consciousness: A Study of the Third Level. J. Conscious. Explorat. Res. 9, 40–61.

[ref32] Lindhard T. (2018b). Levels of Consciousness: The Role of the Heart and Pulsation. Int. J. Soc. Work Hum. Serv. Pract. 6, 65–74.

[ref33] Loevinger J. (1976). Ego Development. Jssey-Bass, San Francisco, CA.

[ref34] MacDonald D. A. Friedman H. L. (2013). “Quantitative Assessment of Transpersonal and Spiritual Constructs” in The Wiley Handbook of Transpersonal Psychology. eds. Friedman H. L. Hartelius G.. 1st ed (Chichester: John Wiley and Sons, Ltd)

[ref35] McCraty R. Atkinson M. Stolc V. Alabdulgader A. A. Vainoras A. Ragulskis M. (2017). Synchronization of human autonomic nervous system rhythms with geomagnetic activity in human subjects. Int. J. Environ. Res. Public Health 14:770. doi: 10.3390/ijerph14070770, PMID: 28703754 PMC5551208

[ref36] McCraty R. Zayas M. A. (2014). Cardiac coherence, self-regulation, autonomic stability, and psychosocial well-being. Front. Psychol. 5:1090. doi: 10.3389/fpsyg.2014.01090 25324802 PMC4179616

[ref37] Mylonas K. Veligekas P. Gari A. Kontaxopoulou D. (2012). Development and psychometric properties of the scale for self-consciousness assessment. Psychol. Rep. 111, 233–252. doi: 10.2466/08.02.07.PR0.111.4.233-252, PMID: 23045866

[ref38] Nasibulina A. (2015). Education for sustainable development and environmental ethics. Procedia Soc. Behav. Sci. 214, 1077–1082. doi: 10.1016/j.sbspro.2015.11.708, PMID: 38324680

[ref39] Patel K.D. (2019). Designing destiny: heartfulness practices to find your purpose and fulfill your potential. Hay House, Inc., Carlsbad.

[ref40] Patel K.D. (2023). Spiritual Anatomy: Meditation, Chakras and the Journey to the Center, Balance, Hachette Book Group. New York.

[ref41] Patel K. D. Pollock J. (2018). The Heartfulness Way New Harbinger Publications, Inc.

[ref42] Pearmain R. O’Connor H. Schenkman M. Jayanna J. Patel K. D. (2023). An exploration of the lived experience of a modern heart-focused meditation practice. J. Transpers. Psychol. 54, 182–198.

[ref43] Portney L.G. (Ed.) (2020). Foundations of Clinical Research: Applications to Evidence-Based Practice, 4e. Philadelphia: FA Davis.

[ref44] Raffone A. Srinivasan N. (2010). The exploration of meditation in the neuroscience of attention and consciousness. Cogn. Process. 11, 1–7. doi: 10.1007/s10339-009-0354-z, PMID: 20041276

[ref45] Ransome Y. (2020). Religion, spirituality, and health: new considerations for epidemiology. Am. J. Epidemiol. 189, 755–758. doi: 10.1093/aje/kwaa022, PMID: 32128570 PMC8059224

[ref46] Sattin D. Magnani F. G. Bartesaghi L. Caputo M. Fittipaldo A. V. Cacciatore M.. (2021). Theoretical Models of Consciousness: A Scoping Review. Brain Sci. 11:535. doi: 10.3390/brainsci11050535 33923218 PMC8146510

[ref47] Savelyeva T. Douglas W. (2017). Global consciousness and pillars of sustainable development: A study on self-perceptions of the first-year university students. Int. J. Sustain. High. Educ. 18, 218–241. doi: 10.1108/IJSHE-04-2016-0063

[ref48] Sparby T. (2015). Investigating the depths of consciousness through meditation. Mind Matter 13, 213–240.

[ref49] Srinivasan N. (2022). Consciousness without content: a look at evidence and prospects. Front. Psychol. 11:1992. doi: 10.3389/fpsyg.2020.01992 PMC7426455 32849160

[ref51] Tanay G. Bernstein A. (2013). State Mindfulness Scale (SMS): development and initial validation. Psychol. Assess. 25, 1286–1299. doi: 10.1037/a0034044, PMID: 24059475

[ref52] Teasdale G. Jennett B. (1974). Assessment of coma and impaired consciousness: a practical scale. Lancet 304, 81–84. doi: 10.1016/S0140-6736(74)91639-0, PMID: 4136544

[ref53] Thiengkamol N. (2011). Development of a model of environmental education inspiration of public consciousness influencing to global warming alleviation. Eur. J. Soc. Sci. 25, 506–514.

[ref54] Thomas C. Holz T. Bhatia S. (2022). Heartfulness Meditation with Yogic Transmission: Creating the spiritual environment for relaxation, wellness and the evolution of consciousness. J. Transpers. Psychol. 54, 168–181.

[ref55] Tripathi V. Bharadwaj P. (2021). Neuroscience of the yogic theory of consciousness. Neurosci. Conscious. 7, 1–15. doi: 10.1093/nc/niab030 PMC8675243 34925910

[ref56] Turner R. G. Carver C. S. Scheier M. F. Ickes W. (1978). Correlates of Self-Consciousness. J. Pers. Assess. 42, 285–289. doi: 10.1207/s15327752jpa4203_10, PMID: 660401

[ref57] van’t Westeinde A. Patel K. D. (2022). Heartfulness meditation: a yogic and neuroscientific perspective. Front. Psychol. 13:806131. doi: 10.3389/fpsyg.2022.806131, PMID: 35619781 PMC9128627

[ref58] Vieten C. Amorok T. Schlitz M. M. (2006). I to We: the role of consciousness transformation in compassion and altruism. J. Relig. Sci. 41, 915–931. doi: 10.1111/j.1467-9744.2006.00788.x

[ref59] Wilber K. (2000). Integral Psychology: Consciousness, Spirit, Psychology, Therapy. Shambhala. Boston, MA.

[ref60] Wilber K. Engler J. Brown D. (1986). Transformations of Consciousness: Conventional and contemplative perspectives on development. Shambhala, Boston, MA.

