# Supplementary material for: Validation of the Awareness Atlas—a new measure of the manifestation of consciousness
Source: Front Psychol. 2024 Mar 21;15:1283980. doi: 10.3389/fpsyg.2024.1283980 (PMC10994143; doi:10.3389/fpsyg.2024.1283980)
Supplement: Supplementary file 1 [file Data_Sheet_1.PDF]

Supplementary Material

Validation of the Awareness Atlas—A New Measure of the Manifestation of Consciousness

Jia Y*, Schenkman M, O Connor H, Jayanna K, Pearmain R, van't Westeinde A, Patel KD

*** Correspondence:** Corresponding Author: joy.y.jia@rutgers.edu

Supplementary Table

Final Scale of the Awareness Atlas

Instructions

Find a quiet place and then answer these questions. It shouldn't take you more than 5-10 minutes. As you read each statement, reflect on how you behave rather than whether you agree or disagree with the statement. To arrive at a ranking, reflect on your experiences over the past two weeks.

Use the following numbers to rank each statement:

1 = Never 2 = Rarely 3 = Occasionally 4 = Frequently 5 = Almost always 6 = Always

As an example, one question states that 'I am receptive to the feelings, needs, and suffering of others.'. You would consider the challenges you accepted and those that troubled you over the past two weeks. Then arrive at a number that reflects your overall level of acceptance. Similarly, for each question, choose an answer that reflects the totality of your experience over the last two weeks. A few of the questions ask about the 'wisdom of the heart'. The term 'wisdom of the heart' refers to the wisdom that arises when you rise above the noise of thoughts, desires, judgment, and emotions.

	OPTIONS FOR RANKING	**Never (1)**	**Rarely (2)**	**Occasionally (3)**	**Frequently (4)**	**Almost Always (5)**	**Always (6)**
**Relationship to Others**							
1	I am receptive to the feelings, needs, and suffering of others.						
2	I notice how others react to me at the time of an interaction.						
3	I notice my reactions to others at the time of an interaction.						
4	I consider the feelings, needs, and suffering of others.						
5	I consider the perspectives of others and learn from them.						
**Listening to the Heart**							
6	I listen to the wisdom of my heart (the wisdom that arises when my preconceived notions, desires, judgment and emotions are silenced) and trust what it tells me.						
7	I feel guided in life, by the wisdom of my heart.						
8	When making decisions and interacting with others, it is easy for me to connect with the wisdom of my heart.						
9	To make decisions in any situation, my heart (inner wisdom) guides me from a place beyond emotion and thought.						
10	I trust my intuition.						
**Connection with Higher Self**							
11	I feel supported by a deeper reality, underlying all of creation.						
12	I feel that I am part of something greater than myself.						
13	I feel a spiritual aspect to my identity, beyond my worldly identity.						
14	I feel that my consciousness is expanding.						

15	I have a feeling of wonder and awe about life.						
16	I have a sense of being one with all beings in the universe.						
**Acceptance and Letting Go**							
17	I cheerfully embrace situations that are hard, uncomfortable, or challenging.						
18	I cheerfully adapt to life circumstances in order to grow.						
19	I embrace all experiences of my life with joy as they unfold.						
20	I accept the struggles and lessons in life.						
21	I use my self-awareness to realize I have choices in how to respond to situations.						
22	My emotions, feelings, and thoughts remain balanced (stable) no matter what is going on within and around me.						
23	As my awareness and consciousness change, I adapt my behaviors in order to be compatible with these changes.						
